# The Clinical Relevance of the NATALEE Study: Application of the NATALEE Criteria to a Real-World Cohort from Two Large German Breast Cancer Centers

**DOI:** 10.3390/ijms242216366

**Published:** 2023-11-15

**Authors:** Henning Schäffler, Franziska Mergel, Kerstin Pfister, Stephan Lukac, Angelina Fink, Kristina Veselinovic, Brigitte Rack, Visnja Fink, Elena Leinert, Moritz Dimpfl, Alexander Englisch, Christian Martin Tegeler, Anna Seller, Eva-Maria Grischke, Markus Hahn, Léa Louise Volmer, Tobias Engler, Marie Louise Frevert, Florin Andrei Taran, Wolfgang Janni, Sara Yvonne Brucker, Andreas Daniel Hartkopf, Dominik Dannehl

**Affiliations:** 1 Department of Gynecology and Obstetrics, University Hospital Ulm, 89075 Ulm, Germany; franziska.mergel@uniklinik-ulm.de (F.M.); angelina.fink@uniklinik-ulm.de (A.F.); elena.leinert@uniklinik-ulm.de (E.L.); wolfgang.janni@uniklinik-ulm.de (W.J.); 2 Department of Gynecology and Obstetrics, University Hospital Mannheim, 68135 Mannheim, Germany; moritz.dimpfl@umm.de; 3 Department of Women’s Health, Tuebingen University, 72076 Tuebingen, Germany; alexander.englisch@med.uni-tuebingen.de (A.E.); christian.tegeler@med.uni-tuebingen.de (C.M.T.); anna.seller@med.uni-tuebingen.de (A.S.); eva-maria.grischke@med.uni-tuebingen.de (E.-M.G.); markus.hahn@med.uni-tuebingen.de (M.H.); lea-louise.volmer@med.uni-tuebingen.de (L.L.V.); tobias.engler@med.uni-tuebingen.de (T.E.); andreas.hartkopf@med.uni-tuebingen.de (A.D.H.); dominik.dannehl@med.uni-tuebingen.de (D.D.); 4 Department of Obstetrics and Gynecology, University of Freiburg, 79106 Freiburg, Germany; marie.louise.frevert@uniklinik-freiburg.de (M.L.F.);

**Keywords:** oncology, breast cancer, systemic therapy, CDK4/6 Inhibitors, Ribociclib, NATALEE

## Abstract

The NATALEE study showed a significant benefit in invasive disease-free survival (iDFS) for patients with HR+/HER2− early breast cancer (eBC) at intermediate and high risk of recurrence who were treated with the CDK4/6 inhibitor Ribociclib in combination with endocrine therapy (ET). This retrospective study aims to apply the NATALEE inclusion criteria to a representative real-world cohort to estimate the proportion of HR+/HER2− breast cancer patients eligible for adjuvant Ribociclib therapy. Patients who underwent full surgical treatment for eBC between January 2018 and December 2020 at two large German university breast cancer centers (University of Ulm, University of Tuebingen) were included. Descriptive statistics were used to characterize the patient population eligible for Ribociclib treatment based on the NATALEE study’s inclusion criteria. Out of 2384 enrolled patients, 1738 had HR+/HER2− eBC, of whom 43% (747/1738) met the NATALEE inclusion criteria. Of note, these patients were older, received less chemotherapy and presented with less advanced tumor stages compared to the NATALEE study cohort. Additionally, compared to the NATALEE study cohort, fewer patients had lymph node involvement (72.4% vs. 88.7%). Our analysis suggests that approximately 43% of all HR+/HER2− breast cancer patients will qualify for Ribociclib treatment. Given the numerous treatment options for patients with HR+/HER2− eBC, as well as the differences between the NATALEE cohort and patients in the real-world clinical setting, future analyses will be needed to determine which patients would benefit most from adjuvant CDK4/6 inhibitor treatment.

## 1. Introduction

In the last few years, the continuous and increasing personalization of therapeutic options in breast cancer treatment has significantly improved prognosis [1,2,3,4]. In advanced or metastatic hormone receptor-positive (HR+) and human epidermal growth factor receptor 2-negative (HER2−) breast cancer, recent studies have demonstrated that the addition of cyclin-dependent kinase 4/6 (CDK4/6) inhibitors to endocrine therapy (ET) resulted in a considerable improvement in progression-free survival (PFS) and overall survival (OS) [5,6,7,8,9,10,11]. CDK4/6 inhibitors, combined with ET, are, therefore, considered the recommended standard of care in the advanced or metastatic disease setting. In the adjuvant setting, it is recommended that patients with HR+/HER2− early breast cancer (eBC) receive ET for at least 5 years [12]. The choice of adjuvant ET mainly depends on menopausal status, as well as the individual risk of recurrence. For low-risk premenopausal patients with HR+/HER2− eBC, Tamoxifen is considered the standard of care [13]. However, for premenopausal HR+/HER2− eBC patients with a high composite risk, analyses of the SOFT and TEXT studies have demonstrated an improvement in 8-year freedom from distant recurrence with exemestane plus OFS versus tamoxifen plus OFS or tamoxifen alone. Postmenopausal patients with HR+/HER2− eBC can be treated in the first five years with AI or sequentially with AI → Tam or Tam → AI [14]. For pre- and postmenopausal patients with an increased risk of recurrence, the individual benefit of extended endocrine therapy should be evaluated [15,16].

In addition to adjuvant ET, patients at an increased risk of relapse might receive adjuvant or neoadjuvant chemotherapy. While there are several risk factors, including age, menopausal status, tumor stage, grading, the expression levels of hormonal receptors (ER and PgR), residual cancer burden, (dynamic) proliferation markers like Ki67, and gene expression analysis, that are useful for guiding decision-making, predicting the exact benefit of chemotherapy for each individual patient remains challenging [17,18,19,20,21,22,23]. To facilitate the identification of high-risk patients and clinical decision-making regarding the escalation or de-escalation of adjuvant therapy, the IRIDE working group has assembled an updated list of relapse risk factors for HR+/HER2− early breast cancer [24].

Moreover, therapeutic options that have proven to be effective in the metastatic setting, e.g., the use of CDK4/6 inhibitors, are increasingly being used in adjuvant treatment for selected, clinically high-risk patients [25,26,27,28]. The MonarchE trial (NCT03155997) investigated the use of adjuvant Abemaciclib in patients with HR+/HER2−, node-positive, and high-risk early breast cancer [27,29]. The study involved 5637 patients and aimed to assess overall survival (OS), invasive disease-free survival (iDFS), and distant relapse-free survival. Patients were randomly assigned to receive standard-of-care ET (ET of physician’s choice) either with or without Abemaciclib. High-risk disease criteria included the presence of four or more positive axillary lymph nodes or between one and three positive nodes with other risk factors. After a median follow-up of 54 months, the five-year efficacy data from the MonarchE trial revealed a hazard ratio of 0.680 (95% CI 0.599–0.772) for invasive disease-free survival (IDFS) and a hazard ratio of 0.675 (95% CI 0.588–0.774) for distant relapse-free survival (DRFS). Numerically, there were fewer deaths in the group that received abemaciclib (208 compared to 234 in the ET-only group), and no new safety concerns emerged. The study suggests that adjuvant abemaciclib reduces the risk of recurrence in high-risk early breast cancer, but further follow-up is needed to determine its impact on overall survival [27]. In contrast, the PALLAS (NCT02513394) [30] and PenelopeB (NCT01864746) [31] trials did not exhibit a significant improvement when using Palbociclib to treat patients with eBC. Recently, the randomized, controlled phase III NATALEE trial evaluated the safety and efficacy of 3 years of Ribociclib treatment (400 mg/day in a 3-week-on, 1-week-off regimen), in combination with adjuvant ET treatment (aromatase inhibitor (AI) for at least 5 years), in patients with HR+/HER2− high and intermediate risk of eBC. Of note is the fact that the NATALEE study protocol allowed the inclusion of patients with both high and intermediate clinical risk, defined as being at Anatomic Stage III or IIB or a subset of Stage IIA (as summarized in the Section 4). After a median follow-up of 27.7 months, significant improvement in iDFS was observed compared to ET alone, with a hazard ratio of 0.75 [95% CI: 0.618–0.906]. Due to an early divergence of iDFS outcomes, the study was prematurely terminated. Recently, the results of the NATALEE trial’s pre-planned exploratory subgroup analysis demonstrated consistent three-year IDFS benefit across all clinical subgroups [32].

In this retrospective analysis, we aimed to apply the inclusion criteria of the NATALEE trial to a representative real-world cohort from two major German university breast cancer centers to model the proportion of patients with HR+/HER2− eBC who could potentially benefit from Ribociclib treatment in a real-world, clinical setting.

## 2. Results

This retrospective analysis encompassed a total of 2384 patients diagnosed with eBC who received complete surgical resection at the Department of Gynecology and Obstetrics, Ulm University Hospital, and the Department of Women’s Health, Tuebingen University Hospital, Germany between January 2018 and December 2020. The most prevalent tumor subtype was HR+/HER2− eBC (72.9%), followed by HER2+ eBC (14.5%) and triple-negative eBC (12.6%). In line with the NATALEE study, the subsequent analysis and discussion will be confined to the cohort of HR+/HER2− patients. Comprehensive patient characteristics for the entire study cohort are provided in Supplementary Table S1. The majority of the 1738 patients with HR+/HER2− eBC were postmenopausal (1203/1738; 69.2%), with an average age of 60.1 years (±12.3). The most common histological type was the non-special type (NST; 1360/1738; 78.2%), followed by invasive lobular carcinoma (ILC; 277/1738; 15.9%). Most patients in this cohort had a small (T1: 1041/1738; 60.1%, T2: 540/1738; 31.1%, T3: 80/1738; 4.6%, T4: 39/1738; 2.0%) or nodal-negative (N0: 1195/1738; 68.8%) tumor. Neoadjuvant chemotherapy was administered to 8.4% (146/1738) of patients, while 18.0% (313/1738) received adjuvant chemotherapy. The majority of HR+/HER2− patients (1279/1738; 73.6%) did not undergo any chemotherapy (See Table 1).

**Figure 1:**
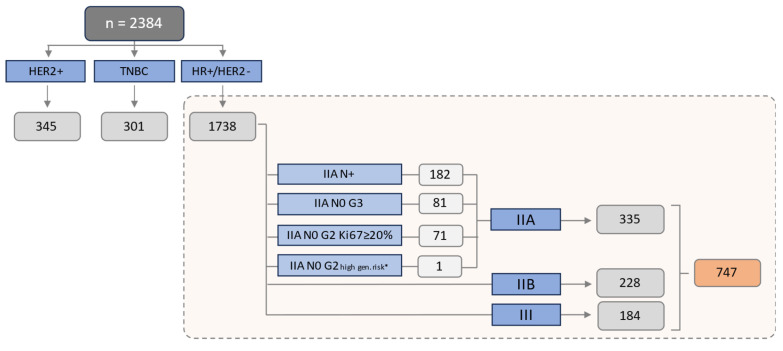
Patients fulfilling the NATALEE inclusion criteria: The study cohort consisted of a total of 2384 patients, comprising 1738 patients with HR+/HER2− eBC, 301 patients with triple-negative eBC (TNBC) and 345 patients with HER2+ eBC. In total, 747 (43.0%) of 1738 patients with HR+/HER2− eBC fulfilled the tumor-specific inclusion criteria of the NATALEE study. IIA: 44.8% (335/747); IIB: 30.5% (228/747); III: 24.6% (184/747). N+: nodal positive; N0: nodal negative. High genomic risk*: Oncotype DX Breast Recurrence Score > 25, or Prosigna/PAM50 categorized as high risk, or MammaPrint categorized as high risk, or EndoPredict EPclin Risk Score categorized as high risk. Staging in line with the UICC/AJCC classification.

The NATALEE inclusion criteria were applied to the HR+/HER2− cohort. Detailed information about the applicable inclusion criteria within this cohort can be found in Figure 1. Consistent with the NATALEE trial, all patients with pathologic lymph node involvement (N+), a tumor size of at least 50 mm (T3 or T4 tumor stage), or a tumor size less than 50 mm, with either G3 Grading or G2 Grading, accompanied by a Ki67 proliferation index ≥ 20%, or with evidence of high genomic risk, were considered eligible. In 8 patients with stage IIA G2 N0, a multigene assay was performed (Onxotype DX). Among these eight patients, one had an RS > 25.

Overall, 43.0% (747/1738) of all HR+/HER2− patients met the inclusion criteria of the NATALEE study. The detailed characteristics of patients potentially eligible for Ribociclib can be extracted from Table 2. These had a mean age of 59.1 years (±13.0) and were predominantly postmenopausal (66.5%), with 66.5% having a tumor size of ≥2 cm, 27.6% (206/747) having nodal-negative disease and 53% having a Ki67 of ≥20%. As per the UICC/AJCC classification, within the cohort of 747 patients fulfilling the NATALEE criteria, 44.8% (239/747) were classified as stage IIA, 30.5% (237/747) as stage IIB and 24.6% (184/747) as stage III. Notably, 50.6% (378/747) of the patients in our real-world cohort did not receive any chemotherapy.

**Figure 2:**
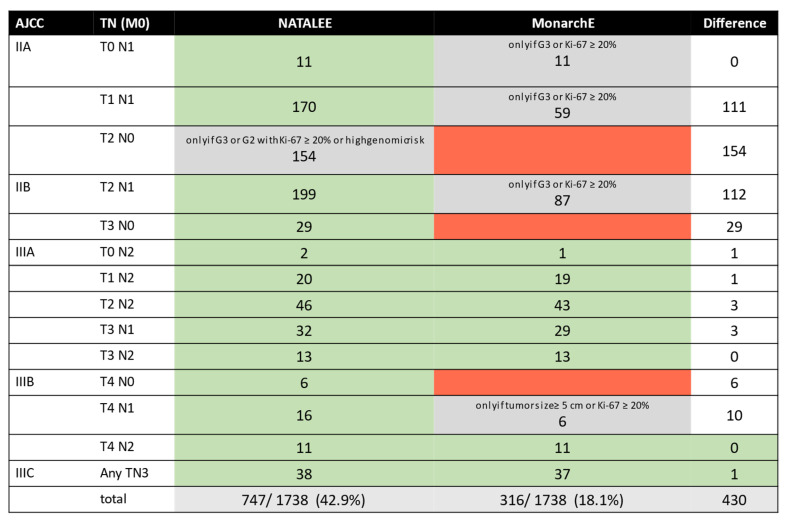
Comparison of patients potentially eligible for Ribociclib versus Abemaciclib therapy analogous with the NATALEE and MonarchE trials: The absolute numbers of potentially eligible patients, based on the inclusion criteria of the NATALEE study versus the MonarchE study, are depicted. According to the study protocols, the NATALEE study primarily considered the post-operative, pathological tumor stage as an inclusion criterion. In the case of neoadjuvant chemotherapy, the clinical TNM stage was also accepted. For Abemaciclib, only the post-operative, pathological TNM stage was considered, in line with the MonarchE study protocol. Fields shaded in gray indicate that the inclusion criteria are met only under specific conditions, as detailed in the table, while fields highlighted in red indicate that the inclusion criteria are not met for that specific tumor stage.

A real-world analysis of the MonarchE trial was previously conducted at the University of Tuebingen [33]. Applying the MonarchE inclusion criteria to this cohort, 18.1% would meet the inclusion criteria. Details and information regarding the overlap of patients eligible for the NATALEE and the MonarchE trials in our real-world cohort are displayed in Figure 2.

## 3. Discussion

Recently, the NATALEE phase III trial achieved its primary endpoint [34]. Adding a 3-year therapy of 400 mg Ribociclib/day to ET with an aromatase inhibitor in patients with intermediate-/high-risk HR+/HER2− eBC resulted in a significant 25.2% relative reduction in iDFS and a significant improvement in distant relapse-free survival (DRFS). Due to the observed early divergence in iDFS outcomes, the study was prematurely terminated, and an extension of the approval of Ribociclib is expected. By applying the NATALEE criteria to a representative real-world cohort, this retrospective analysis provides an estimate of the potentially eligible patient cohort for Ribociclib treatment in the clinical setting. Regarding tumor stage and biology, approximately 43% of all patients met the inclusion criteria of the NATALEE study. There are some notable differences between the cohorts investigated in the NATALEE trial and this real-world analysis. Patients in the real-world cohort were, on average, older, with ages of 59.1 vs. 52.0 years in the NATALEE study, and they exhibited less advanced tumor stages (IIa: 44.8% vs. 18.8%; Stage IIb: 30.5% vs. 20.9%; Stage III: 24.6% vs. 59.9%). Accordingly, the number of patients who received chemotherapy differed, with only 49.4% receiving it in our real-world cohort, compared to 88.2% in the NATALEE trial. Also, there is a notable difference in lymph node involvement, with a significantly higher percentage of nodal-negative patients in the real-world cohort (27.6%) compared to the NATALEE study cohort (11.2%). Of particular note is the disparity in the distribution of tumor stages between the real-world cohort and the NATALEE study cohort, as outlined above. The available analyses of invasive disease-free survival (iDFS) from the NATALEE study demonstrate a significant effect on iDFS with the addition of Ribociclib at both Stage II (HR 0.76; 95% CI 0.53–1.1) and Stage III (HR 0.74; 95% CI 0.59–0.93). However, previous analyses have not outlined any differentiation within the anatomical Stage II subgroup. Further subgroup analyses will be necessary to definitively assess the applicability of the NATALEE study findings in a real-world context.

Even though, formally, 43.0% of the HR+/HER2− patients in this cohort retrospectively meet the NATALEE inclusion criteria, it is to be assumed that in future clinical practice, the proportion of patients treated with Ribociclib will be lower. Factors contributing to this include higher rates of comorbidities, as well as lower patient compliance and therapy adherence in the clinical setting [35]. For example, the ECOG Performance Status rating of 82.6% of patients in the NATALEE study cohort was 0, whereas a median of 1 would be more typical in the real-world setting [36]. In addition to differences in tumor stages, the different rates of chemotherapy can be explained by higher rates of patients who might decline chemotherapy or have contraindications in real-world scenarios [33,36,37].

While therapy with tamoxifen (with or without the use of ovarian function suppression (OFS)) is one of the standard ET treatments for premenopausal patients with HR+/HER2− eBC, only aromatase inhibitor treatment was allowed in the NATALEE study [12,38,39,40]. Of all patients in our real-world cohort potentially eligible for Ribociclib, 32% were premenopausal, making OFS mandatory for these patients [12,32,33,34]. Considering the higher rate of side effects, as well as the higher rate of therapy discontinuation associated with endocrine therapy using an AI plus OFS [41], especially in the intermediate risk cohort, comprehensive patient education and a shared decision-making process are mandatory. In this regard, real-world analyses that focus on the current use of Tamoxifen (with or without OFS) or AI with OFS in premenopausal patients are currently ongoing.

Furthermore, additional, adjuvant treatment options are available for patients with a high individual risk. Adjuvant therapy with Olaparib is available to a small patient population with high-risk HR+/HER2− eBC in the presence of a germline BRCA1/2 mutation [42]. Recently, we were able to demonstrate that approximately 8% of patients with HR+/HER2− eBC meet the clinical–pathological inclusion criteria of the OlympiA study [26,43]. Even though the frequency of a BRCA1/2 mutation is low in the HR+/HER2− cohort, ranging from 1.5% to 5.0%, testing should be strongly recommended in this context, regardless of individual or familial risk, to determine the therapeutic indication for Olaparib [44,45]. Moreover, the CDK 4/6 inhibitor Abemaciclib constitutes a therapeutic option for ER+/HER- eBC patients who meet the clinical high-risk criteria, as defined in the MonarchE trial [27,46]. In line with previous real-world analyses, where 14–19% of patients with HR+/HER2− eBC fulfilled the MonarchE criteria [33,47], our analysis found that 18.1% of the patients fulfilled the inclusion criteria of the MonarchE study and would potentially benefit from Abemaciclib treatment. As shown in Figure 2, there is a significant overlap with the NATALEE cohort.

Genomic tests are employed in HR+/HER2− eBC to safely and individually omit chemotherapy in cases of low risk [17,19,20,21,22]. In patients with HR+/HER2− early breast cancer (eBC) and 0–3 pathologic lymph nodes, the recommendation for chemotherapy can be reduced by approximately 50% through a low-risk Recurrence Score^®^ result using the Oncotype DX^®^ [18]. As therapy with a CDK4/6 inhibitor is also associated with side effects and economic burdens, future studies need to investigate to what extent patients with high clinical but low genomic risk benefit from CDK4/6 inhibitor treatment.

An important limitation of this study is related to its retrospective nature. By conducting it at two major German breast centers, an attempt was made to capture a consistent and representative real-world cohort. However, it can be assumed that the number of patients with stage IIA, G2, and N0 + genomic high risk might be underestimated due to the fact that only eight genomic risk tests were conducted and documented in this retrospective cohort. With the expanding landscape of breast cancer treatment options, the significance of multicenter registries as essential complements to randomized controlled trials (RCTs) is growing. RCTs might not encompass all potential treatment combinations and sequences, making multicenter registries increasingly important for systematically gathering data regarding treatment procedures, clinicopathological risk factors, molecular information and patient outcomes.

## 4. Materials and Methods

This retrospective analysis includes all patients who received full surgical treatment for eBC at the Department of Gynecology and Obstetrics, Ulm University Hospital, and the Department of Women’s Health, Tuebingen University Hospital, in Germany between January 2018 and December 2020. This study was conducted in compliance with the guidelines of the Declaration of Helsinki and approved by the Ethics Committees of both Tuebingen University Hospital (protocol code 296/2023/BO2) and Ulm University Hospital (protocol code 136/23). Alongside complete surgical resection (R0), the inclusion criteria for this retrospective analysis required patients (both female and male) to have no evidence of metastatic disease. Hormone receptor and HER2 receptor expression were evaluated by certified pathologists using local standards. Tumors were classified as HR+ if they showed positive estrogen receptor (ER) and/or progesterone receptor (PR) expression via immunohistochemistry (≥10% for ER, ≥10% for PR). HER2 immunoreactivity was assessed on a scale of 0 to 3+ using the HERCEPT test (DAKO, Glostrup, Denmark). Only tumors with a HER2 score of 3+ or 2+, with detectable HER2 amplification, were considered HER2 positive. HER2 amplification was determined through fluorescence in situ hybridization using the Pathvysion^®^ Kit (Vysis, Downers Grove, IL, USA) in Tuebingen and the ZytoMation^®^ ERBB2/CEN 17 Dual Color FISH Probe (Cytovision GmbH, Bremerhaven, Germany) in Ulm.

The patient selection for potential candidates eligible for Ribociclib was carried out in line with the inclusion criteria of the NATALEE trial [48]: men and pre- or postmenopausal women with histologically confirmed primary invasive HR+/HER2− eBC, as well as complete surgical resection with microscopic margins free of tumors and anatomic stage (AJCC Cancer Staging Manual, 8th edition [49]) III, IIB or IIA (either N1 or N0 with Grade 3 or N0 with Grade 2, and any of the following traits: Ki67 ≥ 20% or evidence of high genomic risk (Oncotype DX RS ≥ 26; Prosigna/PAM50 high risk; MammaPrint or EndoPredict EPclin high risk scores). Patients who underwent neoadjuvant chemotherapy must have met these criteria in any presurgical sample and/or the surgical specimen. Patients with bilateral/multifocal breast cancer must have met these criteria in any of the involved sites.

For the purpose of analyzing the proportion of high-risk patients in our real world cohort who would potentially be eligible for treatment with Abemaciclib, the inclusion criteria of the MonarchE trial were applied: HR+/HER2− lymph-node-positive eBC with either proliferation marker Ki67 ≥ 20% or Ki67 < 20% and (a) at least four pathologic lymph nodes (N2), (b) histologic grade 3 (G3) or (c) a tumor size of at least 50 mm (T3).

Data processing and statistical analysis were conducted using Jupyter Notebook (Version 6.3.0, Project Jupyter, open-access and community-developed) on Anaconda (Version 3.0, Anaconda Inc., Austin, TX, USA) with the Python extension packages pandas (Version 1.4.1, open-access and community-developed) and NumPy (Version 1.22.2, open-access and community-developed). Descriptive statistics were performed, including means and standard deviations. Power Point (Microsoft 365, Microsoft Redmond, Washington, DC, USA) was used for creating flow charts and visualizing the data.

## 5. Conclusions

This retrospective analysis suggests that in a real-world context, approximately 43% of patients with HR+/HER2− eBC could potentially benefit from adjuvant therapy with Ribociclib. This analysis provides a crucial metric for resource planning by presenting, for the first time, a representative estimate of the patient cohort, potentially eligible for Ribociclib treatment in the clinical setting. The applicability and external validity of the safety and efficacy results from the NATALEE trial, as well as the actual number of eBC patients who would receive Ribociclib in routine, clinical practice, needs to be investigated in forthcoming real-world evidence analyses.

## Figures and Tables

**Table 1:** Characteristics of HR+/HER2− patients.

	Number of Patients	Percentage
	1738	100%
**Age**	60.1 ± 12.3	
**Menopausal status**		
Premenopausal	491	28.3
Postmenopausal	1203	69.2
Male	3	0.2
n/a	41	2.4
**Histology**		
NST	1360	78.3
ILC	277	15.9
Other	100	5.8
n/a	1	0.1
**Grading**		
1	214	12.3
2	1247	71.7
3	275	15.8
n/a	2	0.1
T-stage *		
0	43	2.5
1	1041	59.9
2	540	31.1
3	80	4.6
4	34	2.0
N-stage *		
0	1195	68.8
1	415	23.9
2	89	5.1
3	38	2.2
X	1	0.1
**ER status**		
+	1729	99.5
−	9	0.5
**PR status**		
+	1447	83.3
−	291	16.7
**HER2 status**		
+	0	0.0
−	1738	100.0
**Ki67**		
≥20%	580	33.4
<20%	1158	66.6
**Chemotherapy**		
Neoadjuvant	146	8.4
Adjuvant	313	18.0
None	1279	73.6

* T and N stages were assessed after surgery. NST, non-special type; ILC, invasive lobular carcinoma; ER, estrogen receptor; PR, progesterone receptor; HER2, human epidermal growth factor receptor 2; TNBC, triple-negative breast cancer; n/a, not available.

**Table 2:** Characteristics of patients eligible for Ribociclib.

	Number of Patients	Percentage
	747	100%
**Age**	59.1 ± 13.0	
**Menopausal status**		
Premenopausal	240	32.1
Postmenopausal	497	66.5
Male	3	0.4
n/a	7	0.9
**Histology**		
NST	587	78.6
ILC	132	17.7
Other	28	3.7
n/a	0	0
**Grading**		
1	24	3.2
2	508	68
3	215	28.8
n/a	0	0
**T-stage**		
0	32	4.3
1	216	28.9
2	386	51.7
3	80	10.7
4	33	4.4
**N-stage**		
0	206	27.6
1	415	55.6
2	89	11.9
3	37	5
X	0	0
**ER status**		
+	740	99.1
−	7	0.9
**PR status**		
+	607	81.3
−	140	18.7
**HER2 status**		
+	0	0
−	747	100
**Ki67**		
≥20%	399	53.4
<20%	348	46.6
**Chemotherapy**		
Neoadjuvant	129	17.3
Adjuvant	240	32.1
None	378	50.6

NST, non-special type; ILC, invasive lobular carcinoma; ER, estrogen receptor; PR, progesterone receptor; HER2, human epidermal growth factor receptor 2; TNBC, triple-negative breast cancer; n/a, not available.

## Data Availability

The data presented in this study are available on request from the corresponding author. The data are not publicly available to ensure the privacy of sensitive patient information.

## Supplementary Materials

The supporting information can be downloaded via this link: https://www.mdpi.com/article/10.3390/ijms242216366/s1.
