# Supplementary material for: The Clinical Relevance of the NATALEE Study: Application of the NATALEE Criteria to a Real-World Cohort from Two Large German Breast Cancer Centers
Source: Int J Mol Sci. 2023 Nov 15;24(22):16366. doi: 10.3390/ijms242216366 (PMC10671738; doi:10.3390/ijms242216366)
Supplement: Supplementary file 1 [file ijms-24-16366-s001.zip › ijms-2665548-supplementary.pdf]

International Journal of
Molecular Sciences

MDPI

Supplementary Table S1. Patient characteristics for the entire study cohort.

		Percentage
**n**	2384	100
**Age**	59.0 ± 12.8	
**Menopausal status**		
Premenopausal	750	31.5
Postmenopausal	1575	66.1
Male	3	0.1
n/a	56	2.3
**Histology**		
NST	1951	81.8
ILC	301	12.6
Other	127	5.3
n/a	5	0.2
**Grading**		
1	214	9.0
2	1470	61.7
3	694	29.1
n/a	6	0.3
**T-stage**	0	
0	272	11.4
1	1315	55.2
2	657	27.6
3	97	4.1
4	43	1.8
**N-stage**		
0	1712	71.8
1	513	21.5
2	112	4.7
3	46	1.9
X	1	0.0
**ER status**		
+	1971	82.7
-	413	17.3
**PR status**		
+	1614	67.7
-	770	32.3
**HER2 status**		
+	345	14.5
-	2039	85.5
**Ki67**		
≥ 20 %	1126	47.2
< 20 %	1258	52.8
**Chemotherapy**		
Neoadjuvant	505	21.2
Adjuvant	507	21.3
None	1372	57.6

NST, non-special type; ILC, invasive lobular carcinoma; ER, estrogen receptor; PR, progesterone receptor; HER2, human epidermal growth factor receptor 2; TNBC, triple-negative breast cancer; n/a, not available.
